# Metabolomics Reveals Exosome Protection in Aging Mouse Kidneys via Ferroptosis Alleviation

**DOI:** 10.1093/geroni/igaf122.2546

**Published:** 2025-12-31

**Authors:** Yanqiu Xing, Liu Di

**Affiliations:** Qilu hospital of Shandong University, Jinan, Shandong, China (People’s Republic); Shandong University, Jinan, Shandong, China (People’s Republic)

## Abstract

This study evaluated the effects of human umbilical cord mesenchymal stem cell-derived exosomes (hUCMSC-exos) on renal function in naturally aging mice and explored underlying mechanisms. Twenty-four-month-old C57BL/6J mice were divided into a natural aging group (WT-AC, n = 10) and an hUCMSC-exos intervention group (WT-AEX, n = 10). Blood and kidney tissues were analyzed for biochemical markers, histological changes, and metabolomic profiles. hUCMSC-exos significantly reduced blood urea nitrogen, creatinine, and uric acid levels compared to the natural aging group (P < 0.01). Histological examination revealed reduced inflammatory cell infiltration, tubular degeneration, and interstitial fibrosis in the hUCMSC-exos group. Transmission electron microscopy demonstrated mitochondrial shrinkage, cristae rupture, and podocyte foot process effacement in the natural aging group, while the hUCMSC-exos group exhibited restored mitochondrial morphology, including intact cristae and reduced swelling. Aging markers (SA-β-Gal, p16INK4a, and γH2AX) were downregulated in the hUCMSC-exos group. Metabolomics identified 62 differential metabolites, with indoxyl sulfate as a potential biomarker for age-related renal injury. Enriched pathways included glutathione metabolism, glycerolipid metabolism, and aminoacyl-tRNA biosynthesis. hUCMSC-exos inhibited ferroptosis by reducing divalent iron ions and malondialdehyde levels, increasing the glutathione/oxidized glutathione ratio, and upregulating glutathione peroxidase 4 expression. These findings suggest that hUCMSC-exos improve renal function, reduce inflammation and fibrosis, and mitigate aging-related structural changes in naturally aging mice. The study highlights the protective role of hUCMSC-exos against renal aging through modulation of key metabolic pathways and ferroptosis inhibition, providing potential therapeutic targets for aging-related kidney injury.

